# Evaluating the Role of the Renin-angiotensin System in COVID-19: Implications for ACE Inhibitor and ARB Use During SARS-CoV-2 Infection

**DOI:** 10.33696/immunology.6.213

**Published:** 2024

**Authors:** Sarah R. Tritsch, Evelyn Mendoza-Torres, Mónica Gómez-Pulido, Jairo Castellar-López, Rebecca Lynch, Carlos Herrera Gomez, Hana Akselrod, Adrienne Poon, Sam Simmens, Christopher N. Mores, Gary Simon, Lauren C. Ray, Sarah Conway, Aileen Y. Chang

**Affiliations:** 1 George Washington University, Washington, D.C., 20037, USA; 2 Faculty of Health, Exacts and Natural Sciences, Universidad Libre, Barranquilla, Colombia; 3 University of the Incarnate Word, San Antonio, TX, USA

**Keywords:** Cardiovascular disease, Coronavirus disease, Diabetes, Hypertension, COVID-19

## Abstract

This study aimed to investigate the role of the renin-angiotensin system (RAS) in COVID-19, particularly focusing on key components such as ACE, ACE2, and their related peptides, angiotensin-(1–7) and angiotensin-(1–9). Using serum samples from healthy controls and both non-severe and severe COVID-19 patients, ELISA assays revealed no significant differences in these RAS components between the groups. In addition, *in vitro* studies showed no impact of ACE inhibitors or Angiotensin Receptor Blockers (ARB) on cell viability during SARS-CoV-2 infection. These clinical findings suggest that RAS alterations may not be a major factor in COVID-19 severity and the *in vitro* data support current guidelines, indicating the safety of continuing ACE inhibitors and ARBs in COVID-19 patients without evidence of increased SARS-CoV-2 infectivity in the presence of these compounds. This study highlights the lack of significant changes in key RAS components during COVID-19 in a clinical cohort and provides critical *in vitro* evidence supporting the continued use of ACE inhibitors and ARBs in treating patients.

## Introduction

Patients with cardiovascular disease, diabetes, and hypertension have high mortality rates in the setting of coronavirus disease 2019 (COVID-19) [1]. Patients with these comorbidities are commonly treated with renin angiotensin system blockers, such as angiotensin-converting enzyme inhibitors (ACEIs) or angiotensin receptor blockers (ARBs) [2–4]. Currently, the use of ACEIs/ARBs in patients with COVID-19 or at risk of COVID-19 infection is currently a subject of intense debate [5–7].

SARS-CoV-2 uses the angiotensin-converting enzyme 2 (ACE2) as receptor for entry into target cells of the lung, intestine, kidney, heart, and blood vessels [8–10]. Both ACE and ACE2 belong to the ACE family of dipeptidyl carboxydipeptidases but exert opposing physiological functions [11,12]. ACE cleaves angiotensin I to angiotensin II, which in turn binds and activates angiotensin II type 1 receptor (AT1R) [11] This activation leads to vasoconstrictive, proinflammatory, and pro-oxidative effects [13,14]. In contrast, ACE2 also degrades angiotensin II to angiotensin-(1–7) and angiotensin I to angiotensin-(1–9) leading to anti-inflammatory, antioxidative, cardioprotective, and vasodilatory effects [11–15].

ACE2 expression is elevated in patients taking ACEIs/ARBs leading to speculation that taking these medications may facilitate COVID-19 infection [1,16,17]. However, ACEIs/ARBs are used for the treatment of patients with common comorbidities such as hypertension and diabetes and are hospitalized with COVID-19 [6]. A recent study showed that angiotensin II induces reactive oxygen species, DNA damage, and T-cell apoptosis in severe COVID-19 [18] and Liu et al. found that serum angiotensin II levels in patients with COVID-19 pneumonia were significantly higher compared with healthy individuals and were linearly associated with viral load and lung injury [19]. Based on this, it can be postulated that SARS-CoV-2 binding to ACE2 may attenuate ACE2 activity, skewing the ACE/ACE2 balance to a state of heightened angiotensin II activity leading to acute lung injury via pulmonary vasoconstriction and inflammatory and oxidative organ damage. Conceivably, renin angiotensin system modulation, either by ACEIs/ARBs, recombinant ACE2, or angiotensin-(1–7) or (1–9), leading to increased expression of ACE2 may help mitigate some of these deleterious effects of angiotensin II. It is also postulated that increased levels of soluble form of ACE2 may act as a competitive interceptor of SARS-CoV-2 and slow virus entry into the cells and protect from lung injury [1].

There is recent clinical data on the utility of initiating ACEI/ARB therapy in patients with COVID-19 [7,20,21]. Pan *et al*. found that patients treated with ACEI/ARB during their hospitalization for COVID-19 showed lower levels of soluble urokinase plasminogen activator receptor and C-reactive protein [20]. However, the role of ACE2, vasoactive peptides (angiotensin-(1–7) and (1–9)) and ACEI/ARBs in SARS-CoV-2 infection is not well understood but determination of the appropriate use of these medications in the management of SARS-CoV-2 patients is critical. In this article we report the results of an analysis of serum levels of angiotensin-(1–7) and (1–9) and ACE and ACE2 from samples of patients with non-severe and severe COVID-19 compared to non-infected controls and the use of the novel peptides angiotensin-(1–7) and (1–9) in preventing cell death in the setting of COVID infection in an *in vitro* model.

## Materials and Methods

### Setting

Participants over the age of 18 with acute COVID-19 infection diagnosed via RT-PCR at 1–11 days of symptom onset and hospitalized at George Washington University (GWU) Hospital were recruited from January 2020 to December 2021 during the pandemic.

### Ethics statement

All participants received written informed consent to donate blood samples to the Coronavirus Specimen Bank under the GWU Institutional Review Board approved protocol #NCR202387.

### Biosafety

All studies with samples from COVID-19-infected patients were performed in certified BSL-3 laboratories in biological safety cabinets and were conducted under the approved GWU Institutional Biosafety Committee protocol #IBC-20–060.

### Outcome measures

Participants were enrolled during the febrile phase and symptom assessment, including assessment of the COVID-19 signs and symptoms, and hospital course was recorded from the patient self-report and chart review.

### Case classification

Clinical COVID-19 is classified into four levels based on the severity of symptoms: mild, moderate, severe, and critical as per the National Institutes of Health (NIH). NIH defined the clinical spectrum of disease as:

- **Asymptomatic or presymptomatic infection:** Individuals who test positive for SARS-CoV-2 using a virologic test (i.e., a nucleic acid amplification test or an antigen test) but who have no symptoms that are consistent with COVID-19.
- **Mild illness:** Individuals who have any of the various signs and symptoms of COVID-19 (e.g., fever, cough, sore throat, malaise, headache, muscle pain, nausea, vomiting, diarrhea, loss of taste and smell) but who do not have shortness of breath, dyspnea, or abnormal chest imaging.
- **Moderate illness:** Individuals who show evidence of lower respiratory disease during clinical assessment or imaging and who have saturation of oxygen (SpO2) ≥ 94% on room air at sea level.
- **Severe illness:** Individuals who have SpO2 <94% on room air at sea level, a ratio of arterial partial pressure of oxygen to fraction of inspired oxygen (PaO2/FiO2) <300 mm Hg, respiratory frequency >30 breaths/min, or lung infiltrates >50%.
- **Critical illness:** Individuals who have respiratory failure, septic shock, and/or multiple organ dysfunction.

For the purposes of this analysis asymptomatic, mild and moderate cases were grouped into the “non-severe” category and severe and critical cases were grouped into the “severe” category. Non-infected controls were specimen bank donors with no symptoms, prior to COVID vaccination and self-reported adherence to social distancing without known exposures [22].

### Sample collection

After informed consent, forty milliliters of blood were collected for plasma, serum, and peripheral blood mononuclear cell banking at GWU.

### Data management

All patients were assigned a unique patient identification number, which was used in the database and for patient sample labeling. All patient data was void of personal identifiers and was stored in the REDCap database at GWU.

### Enzyme-linked immunosorbent assay (ELISA) analysis

Commercially available ELISA kits were used to quantitatively measure the concentration of human ACE, ACE2, Angiotensin-(1–7) and Angiotensin-(1–9) in serum samples from healthy controls and severe and non-severe COVID patients. The ACE and ACE2 ELISA kits were manufactured by RayBiotech (Norcross, GA, USA) and the Angiotensin-(1–7) and (1–9) ELISA kits were from Biomatik (Willmington, DE, USA). Standard curves were performed to determine the concentration of the target proteins in patient samples. Standards and samples were performed in duplicate.

### Virus

SARS-CoV-2 virus (lineage B.1.617.2, Delta variant, NR-55611) was obtained from BEI Resources (Manassas, VA, USA). The virus was grown by adding 100 ul of the virus to a flask of Vero E6 (African green monkey kidney) cells (CRL-1586; ATCC, Manassas, VA, USA) in serum free Dulbecco’s Modified Eagle Medium (DMEM) containing 1X antibiotic-antimycotic solution and L-glutamine. After incubation at 37°C and 5% CO_2_ for 48 hours, the supernatant was collected and centrifuged at 500 × g for 5 minutes to remove cellular debris. Virus was then aliquoted and stored at −80°C. Cells were infected with a multiplicity of infection (MOI) of 0, 0.1, 0.5, 1.

### Immunofluorescence assay

Vero E6 cells containing high levels of endogenous ACE2 (Vero E6-ACE2, BEI Resources, Manassas, VA, USA) were maintained in DMEM containing 10% fetal bovine serum (FBS) and L-glutamine. Confluent Vero E6-ACE2 cells plated in clear-bottom 96-well plates were infected with SARS-CoV-2 either 24 hours before, 24 hours after, or during exposure to ACEI (lisinopril, 24, 60 or 80 ng/ml), AT1R (losartan, 100, 200, 600, or 800 ng/ml) and AT2R blockers (angiotensin-(1–7) and angiotensin-(1–9), 2 nM, 20 nM, 0.1 uM or 1 uM).

One set of plates was treated with the drugs and incubated for 24 hours, then media was removed and new media containing virus and drugs at appropriate concentrations was added to each well and incubated for 48 hours. A second set of plates was infected with virus, incubated for 24 hours, then media was removed and new media containing drugs at appropriate concentrations was added to each well, and incubated for 48 hours. The third set of plates was treated with the virus and drugs at the same time, then incubated for 48 hours. (The first iteration of the IFA experiment had a final incubation of 24 or 72 hours in addition to 48 hours. This data can be found in the supplemental files.) All live cell incubations were performed in a humidified incubator at 37°C and 5% CO_2_.

After the final incubation, plates were fixed in 4% formaldehyde in phosphate buffered saline (PBS) at 4°C overnight, then washed once with PBS and stained with Hoechst nuclear stain at a 1:5000 dilution. Cell counts were assessed using nuclear morphology on a Biotek Lionheart Lx fluorescent microscope utilizing a 4x objective.

### Statistical methods

For comparing protein levels (ACE, ACE2 etc) between non-infected and non-severe COVID-19 cases and severe COVID-19 cases, an exact version of the Wilcoxon Rank-Sum Test was used which incorporated Monte Carlo estimates and pairwise test using the Dwass, Steel, Critchlow-Fligner Method (SAS version 9.4; SAS/STAT 15.3). This non-parametric approach allowed incorporation of values below and above limits of detection. The sample sizes were n=10 non-infected, n=14 non-severe COVID-19 cases, and n=16 severe covid cases. Statistical power can be approximated through power calculations assuming a t-test comparing the 2 small groups. With n=10 and n=14 in the two groups, there is 83% power to detect a standardized effect size of 1.1 (alpha=.05, 2-sided test). This is typically conisered a large effect size.

For the experiments comparing drug effects on the immunofluorescence assay results, three-way ANOVA models were used to simultaneously test the effect of drug concentration, timing, and MOI. Data were averaged over well replicates. Data were approximately normally distributed.

## Results

### Patient characteristics and outcomes

The age and gender of participants are shown in Table 1. COVID-19 cases were older than non-infected participants. Among the COVID-19 cases, the severe cases were older than non-severe cases and male sex was slightly more common in severe vs. non-severe cases. COVID-19 cases were predominantly African-American, with one Hispanic participant in the non-severe group and one in the severe group while the non-infected participants included Caucasian, African American, Hispanic, and Asian. Descriptive statistics on symptoms and outcomes for the two COVID-19 severity groups are presented in Tables 2 and 3. The most common COVID-19 symptoms among non-severe and severe cases were vomiting, diarrhea, myalgias, and fever. Among the non-severe cases, approximately one-third required nasal cannula oxygen support and the median duration of hospitalization was 3.5 days. In contrast, among severe cases, there were two deaths, all but one case required ventilatory support, including three cases with intubation, and the hospitalization duration was a median of 9 days.

### RAS components in healthy and COVID-19 patients

**Figure 1.**
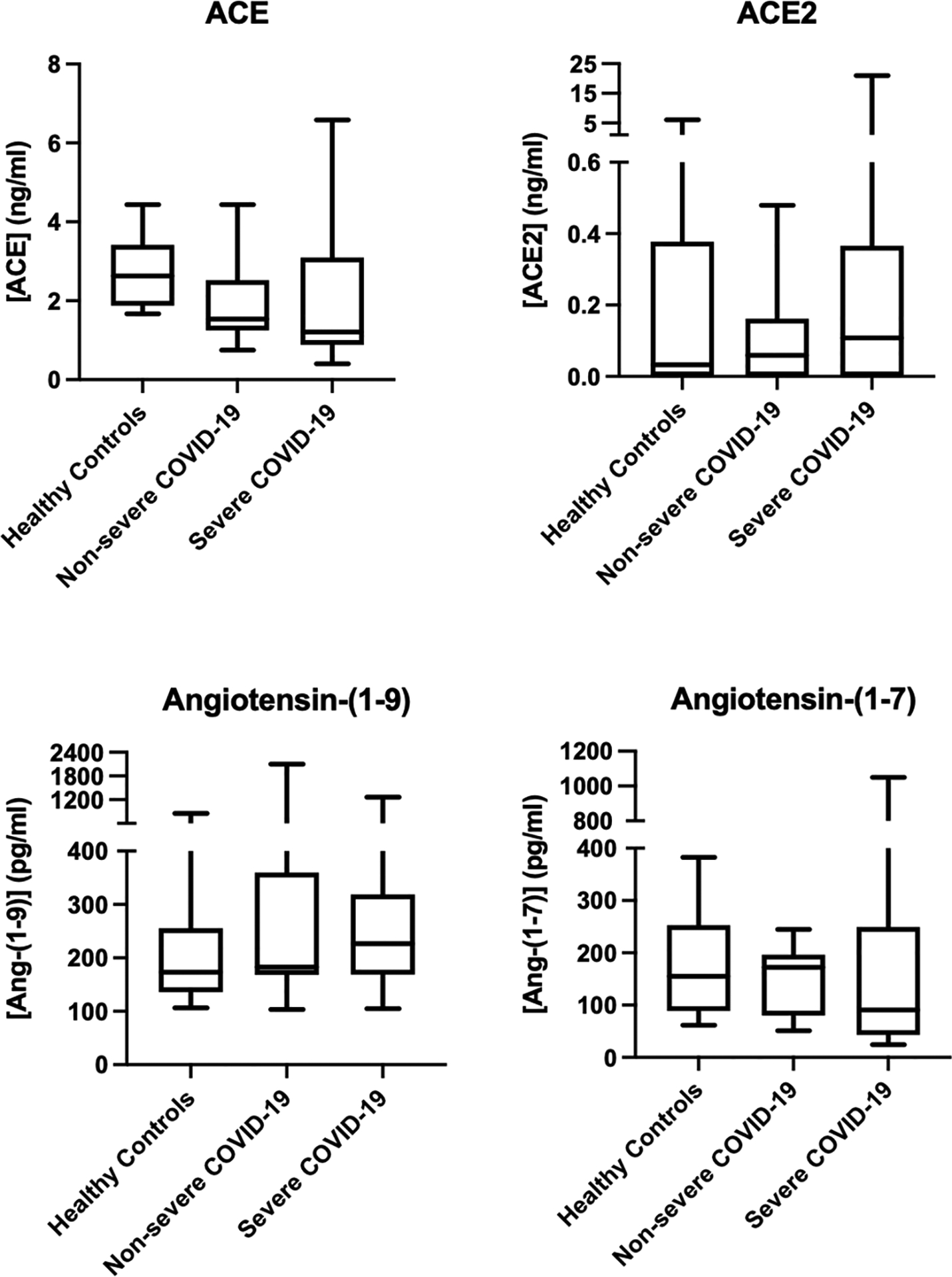
Comparison of the median concentrations of ACE, ACE2, Angiotensin-(1–9) and Angiotensin-(1–7) determined by ELISA in serum samples of healthy controls and non-severe and severe COVID-19 cases. Median and interquartile ranges are shown.

ELISAs were performed using serum samples from healthy controls and non-severe or severe COVID-19 patients. There was no a significant difference in serum levels of ACE, ACE2, Angiotensin-(1–9), and Angiotensin-(1–7) when comparing healthy controls to all COVID-19 cases, or when comparing healthy controls, non-severe COVID-19 cases, and severe COVID-19 cases to each other (Figure 1).

### Comparison of RAS protein levels by clinical outcome

Concentrations of the RAS proteins (ACE, ACE2, Angiotensin1–7, and Angiotensin1–9) did not differ based on clinical outcomes. Concentrations of the RAS proteins were compared between patients with any critical illness event (including respiratory failure, acute respiratory distress syndrome, circulatory shock, liver function test abnormalities, coagulopathy, encephalopathy, myocarditis, and testicular damage) to patients without any critical illness event (Table 4). No group differences were statistically significant. Furthermore, concentrations of the RAS proteins were not correlated with length of hospital stay among the hospitalized COVID-19 cases (Table 5). ICU admission and death were rare events such that statistical analysis is not meaningful.

### Drug type and concentration do not impact live cell numbers

**Figure 2.**
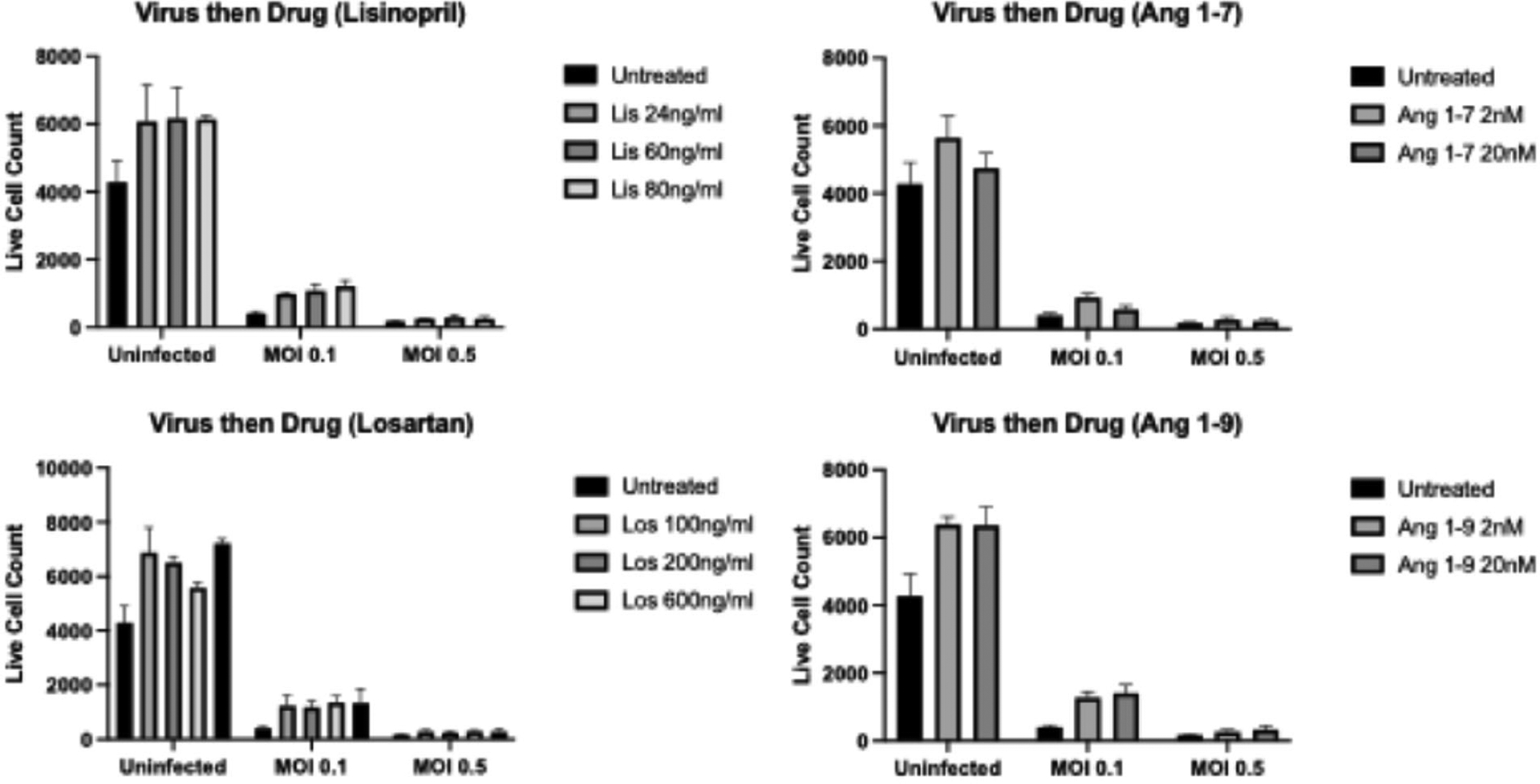
Average cell count via IFA for samples infected with SARS-COV-2 for 24 hours at 0.1 and 0.5 MOI, followed by drug treatment for 48 hours.

**Figure 3.**
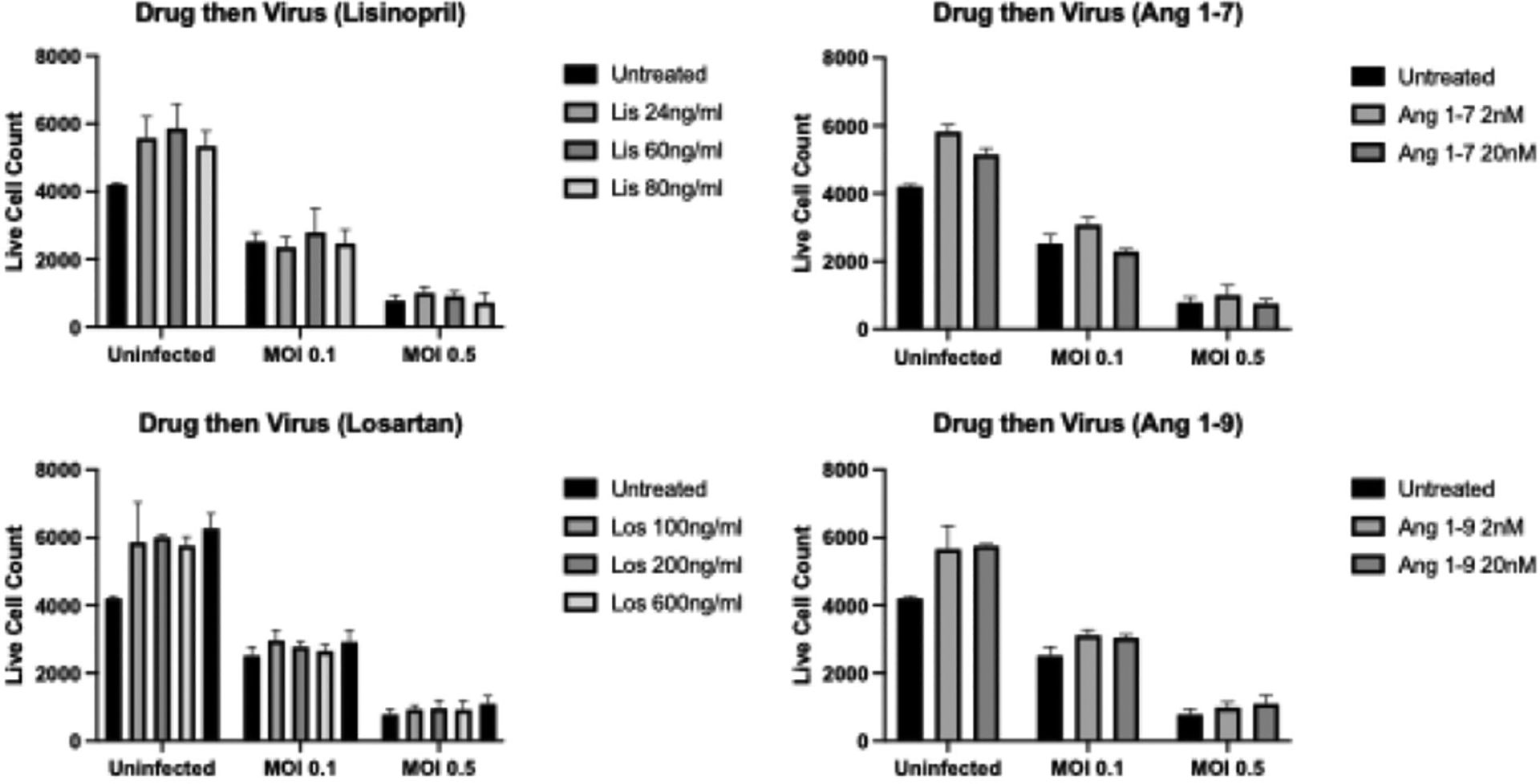
Average cell count via IFA for samples treated with drugs for 24 hours, followed by SARS-COV-2 infection for 48 hours at 0.1 and 0.5 MOI.

**Figure 4.**
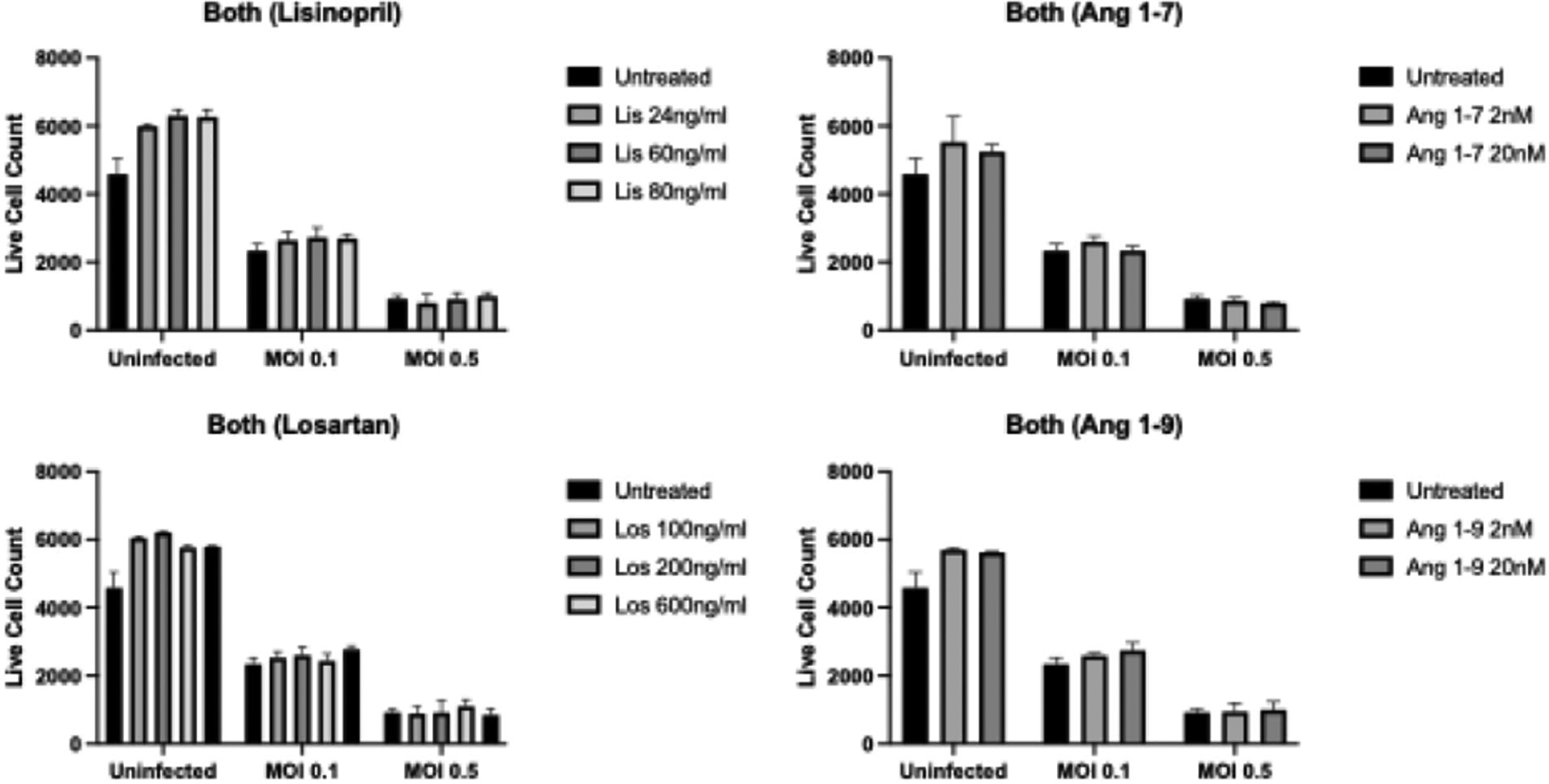
Average cell count via IFA for samples infected with SARS-COV-2 at 0.1 and 0.5 MOI and treated with drugs at the same time for 48 hours.

Immunofluorescence assays (IFAs) were performed to determine whether ACEI/ARBs cause cell death when delivered before, after, or during COVID-19 infection. Delivery timing (virus first, drug first, together) and MOI (0, 0.1, 0.5) were significantly associated with differences in live cell numbers, as was expected (p<0.001 for each). However, there was no association between drug type/concentration and live cell numbers (p>0.05). Figures 2–4 display representative data from the IFA experiments, where samples were infected with 0.1 or 0.5 MOI for 24 hours, followed by drug treatment for 48 hours (Figure 2); were treated for 24 hours, followed by infection for 48 hours (Figure 3); or were simultaneously treated and infected for 48 hours (Figure 4).

## Discussion

The objectives of this study were to determine the differences in protein levels related to the RAS in healthy controls and COVID-19 cases and to assess the role of ACE-inhbition, angiotensin receptor blockade and supplementation with angiotensin peptides involved in the RAS in cell death in the setting of *in vitro* SARS-CoV2 infection at differing conditions of drug delivery timing, concentration and viral concentration (MOI). The ELISA assays revealed no significant differences between ACE, ACE2, Ang (1–9), or Ang (1–7) when comparing healthy controls with COVID-19 cases or among COVID-19 severity subgroups. These results align with recent findings, which suggest variability in RAS component levels in COVID-19 patients [23]. This may be attributed to the heterogeneous nature of COVID-19 and the complex interplay of RAS components in different patient populations [24]. This data further suggests that ACE alone may not be a reliable biomarker for COVID-19 severity in our cohort, a finding that is in line with previous research. Additionally, this data correlates with the findings by Semenzato *et al*. (2021), who did not find a strong correlation between ACE levels and COVID-19 severity, which supports our observation that ACE levels may not directly correlate with disease outcomes [25]. Furthermore, Osman *et al*. (2021) observed that while RAS components were modulated in COVID-19 patients, their levels did not consistently correlate with clinical severity [26] and, in a recent study by Kakavandi *et al*. (2024), the levels of ACE2 and Ang II in SARS-CoV-2 positive and negative subjects were not significantly different [27]. Therefore, our study finding no significant differences in protein levels related to the RAS between healthy controls and COVID-19 cases aligns with previous research suggesting that RAS component levels alone may not be a reliable biomarker for COVID-19 severity.

On the other hand, our results related to ACE2 levels in patients with COVID-19 are discordant with a recent study by Shaheen *et al*. (2024), who found that ACE2 concentration was higher in COVID-19 patients compared to COVID-19 cured and healthy controls [28]. Although they also used a small sample and also used ELISA, the differences may be due to the type of population studied. Our results are also discordant with the study by Silva *et al*. (2022) in Argentina, in fact, in their study, patients with COVID-19 showed higher levels of ACE2 compared to healthy patients Ang-(1–7) and Ang II were significantly lower [29] and the study by Ghimire *et al*. (2023) in which Ang 1–7 levels were significantly lower, whereas Ang II levels were higher in the COVID-19 patients than in healthy control individuals [30]. Differences in the methodology used to quantify RAS components, the sample size, and the race of the patients studied could explain the discordances.

It is important to note that our non-infected population differed from our infected population in age and ethnicity, which could introduce confounding variables. However, this aligns with previous reports showing COVID-19 severity risk increases exponentially with age [31]. COVID-19 rates and severity were also higher for members of ethnic minority groups compared to Caucasian populations [32].

A limitation of our study is the small sample size, which only provided good statistical power to detect large effect sizes. Similar limitations have been reported in other studies examining RAS components in COVID-19 [33,34]. Larger studies incorporating more diverse patient populations and experimental models, will be crucial to advance our understanding of RAS components in COVID-19 and to inform potential therapeutic strategies. Temporal variations in COVID-19 infection could be captured using a longitudinal analysis to investigate changes in RAS components at the acute, progressive, and recovery stages of infection. Furthermore, multivariable analyses and/or stratification by medication use and comorbidities were not planned because the small sample size would result in under-powered significance tests with unreliable estimates. A larger study would be needed to address adjustment for these potential confounders. In addition, *in vitro* testing can not replicate the complexities found *in vivo*, further testing in animal models or clinical trials is needed to validate the effects of RAS inhibitors in COVID-19 patients. Moreover, including additional cell types such as pulmonary cells and cardiomyocytes in experimental models could offer a more comprehensive understanding of the cellular mechanisms underlying ACE and other RAS component levels in COVID-19 [35,36]. Analysis of inflammatory pathways active during COVID-19 may affect or be affected by the RAS system and would be an interesting area for future research. For instance, according to Gavin *et al*. the intersection of bradykinin signaling with the RAS pathway during COVID-19 infection could contribute to vasodilation and vascular permeability [37]. Additionally, examining a broader range of experimental conditions and patient populations could provide deeper insights into the relationship between RAS components and COVID-19 severity [38,39].

Particularly, the potential role for Ang (1–7), a peptide known for its anti-inflammatory and vasodilatory effects, should be further investigated in larger cohorts. Seyedmehdi *et al*. (2022) and Luna *et al*. (2022) have shown that Ang (1–7) could modulate inflammatory responses and improve outcomes in various conditions [39,40]. The trend observed in our study suggests that the role of Ang (1–7) in disease modulation should be further investigated, echoing findings from Maranduca *et al*. (2022) and Valle Martins *et al*. (2021), who noted its potential therapeutic benefits in COVID-19 [38,41].

Interestingly, our *in vitro* study did not find a significant correlation between drug type (ACE-inhibitor, ARB, Ang 1–7 or Ang 1–9) or concentration and the number of viable cells during SARS-CoV2 infection. This data emphasizes that drug type alone may not entirely explain the variance in patient reactions. This supports the findings of Dambha-Miller *et al*. (2022) and Hakeam *et al*. (2021), who revealed varying effects of RAS inhibitors on COVID-19 outcomes [42,43]. Furthermore, despite some reports that ACEI/ARB drugs put COVID-19 patients at high risk for moderate to severe forms of COVID-19, our *in vitro* data support the American Heart Association guidelines to continue RAS inhibitors during COVID-19 as we did not identify an increase in infectivity or cell death with varying concentrations of RAS inhibitors [44].

In conclusion, our study found no significant differences in protein levels related to the RAS between healthy controls and COVID-19 cases nor among clinical outcomes, aligning with previous research suggesting that RAS components, such as ACE, may not be reliable biomarkers for COVID-19 severity. Importantly, our *in vitro* findings that ACE inhibition and angiotensin blockade does not appear to affect SARS-CoV-2 infectivity or increase cell death further support the American Heart Association’s recommendations to continue RAS inhibitors in COVID-19 patients however larger clinical studies are needed to assess this effect in clinical populations. While these results contribute to the safety of RAS inhibitors during COVID-19, further investigation is needed to explore the potential role of Ang (1–7) in modulating disease outcomes and to better understand the complex interactions of RAS components in different populations and conditions.

## Figures and Tables

**Table 1.** Age and gender of participating patients.

	Non-infected n=10	Non-severe COVID-19 case n=14	Severe COVID-19 case n=16
Median Age in Years (SD)	27.5 (5.3)	49 (10.9)	58 (9.5)
% Female Sex	50.0%	42.9%	37.5%
Ethnicity			
Caucasian	50.0%		
African-american	10.0%	92.9%	93.8%
Hispanic	10.0%	7.1%	6.3%
Asian	30.0%		

**Table 2.** COVID-19 signs and symptoms of the participants in this study.

Symptom	Non-severe COVID-19 case n=14	Severe COVID-19 case n=16
Fever	4 (28.6%)	8 (50.0%)
Headache	1 (7.1%)	3 (18.8%)
Arthralgias	1 (7.1%)	2 (12.5%)
Myalgias	5 (35.7%)	7 (43.8%)
Rash	0 (0.0%)	1 (6.3%)
Abdominal Pain	4 (28.6%)	3 (18.8%)
Vomiting	7 (50.0%)	4 (25.0%)
Diarrhea	5 (35.7%)	3 (18.8%)
Somnolence	0 (0.0%)	1 (6.3%)
Hypotension	0 (0.0%)	1 (6.3%)
Hepatomegaly	0 (0.0%)	1 (6.3%)

**Table 3.** Outcomes of the participating patients.

Outcome	Non-severe COVID-19 case n=14	Severe COVID-19 case n=16
% Death	0 (0%)	2 (12.5%)
Ventilation	5 (35.7%)	15 (93.8%)
Nasal Cannula	5 (35.7%)	11 (68.8%)
High flow nasal cannula	0 (0%)	8 (50.0%)
Intubation	0 (0%)	3 (18.8%)
% Intensive Care	2 (14.3%)	3 (18.8%)
Median (IQR) days hospitalized	3.5 (IQR 3.75)	9 (IQR 10)

**Table 4.** RAS Protein Concentration by Any Critical Illness Event. Any critical illness event includes respiratory failure, acute respiratory distress syndrome, circulatory shock, liver function test abnormalities, coagulopathy, encephalopathy, myocarditis, and testicular damage.

RAS protein	With any critical illness event *Median (Q1, Q3)*	Without any critical illness event *Median (Q1, Q3)*	p*
ACE	1.26 (0.94, 1.80)	1.58 (1.11, 3.48)	0.28
ACE2	0.18 (0.01, 0.37)	0.13 (0.01, 1.81)	0.50
Angotensin (1–7)	80.47 (46.34, 457.78)	172.24 (85.40–208.18)	0.48
Angotensin (1–9)	214.03 (161.28, 224.10)	195.60 (171.86, 334.60)	1.0

* Wilcoxon Two-Sample Test

**Table 5.** Pearson correlation of RAS proteins with length of hospital stay among the hospitalized COVID-19 cases.

RAS protein	Pearson correlation coefficient	p
ACE	−0.07	0.73
ACE2	−0.16	0.40
Angotensin (1–7)	−0.08	0.66
Angotensin (1–9)	−0.07	0.70
